# Identification of metabolites of dalfampridine (4-aminopyridine) in dog and rat

**DOI:** 10.3109/21556660.2013.794143

**Published:** 2013-04-12

**Authors:** Anthony Caggiano, Andrew Blight, Tom J. Parry

**Affiliations:** Acorda Therapeutics Inc., Ardsley, NYUSA

**Keywords:** 4-aminopyridine, Dalfampridine, Dog, Metabolites, Preclinical models, Rat

## Abstract

**Background:**

Dalfampridine (4-aminopyridine; 4-AP) is a potassium channel blocker available in the United States to improve walking in patients with multiple sclerosis as demonstrated by an increase in walking speed. Its pharmacokinetics have been evaluated in human studies but its metabolites are not well characterized. This study characterizes the metabolic profile of dalfampridine in two animal species that were used to support nonclinical toxicology evaluation.

**Methods:**

Metabolic profiling of single oral ^14^C-4-AP doses was performed in 12 adult male Sprague–Dawley rats. Similarly, metabolic profiling was performed in beagle dogs in two studies that administered ^14^C-4-AP by gastric intubation; the first study included six animals (three males, three females), and the second study included two animals (one male, one female). Blood and urine samples were evaluated using high performance liquid chromatography, thin layer chromatography, and radioanalysis (liquid scintillation counting), with further identification of components by gas chromatography/mass spectrometry.

**Results:**

Five radioactive components, M1–M5, were detected in rat plasma, although most of the radioactivity corresponded with unchanged 4-AP. Based on R_f_ values, M1 and M2 coseparated with reference standards of 3-hydroxy-4-AP and 4-AP, respectively. Additionally, components M1, M2, and M3 coseparated with the same components isolated from the urine of a dog dosed with ^14^C-4-AP and identified as 3-hydroxy-4-AP, 4-AP, and 3-hydroxy-4-AP sulfate, respectively; M4 and M5 could not be identified because of low concentrations. In dogs, most of the radioactivity was excreted within the first 24 hours as unchanged compound.

**Conclusions:**

Following oral dosing, 4-AP was rapidly absorbed in rats and dogs, with rapid excretion and almost complete urinary recovery in dogs. The primary metabolites in both animal models were 3-hydroxy-4-AP and 3-hydroxy-4-AP sulfate. Systemic clearance not accounted for by renal excretion of 4-AP may occur by liver metabolism by hydroxylation of 4-AP to 3-hydroxy-4-AP followed by sulfate conjugation to 3-hydroxy-4-AP sulfate.

## Introduction

Dalfampridine is the United States nonproprietary drug name for the chemical 4-aminopyridine (4-AP), a potassium channel blocker. It is available as dalfampridine extended release tablets (dalfampridine-ER; known as prolonged-release fampridine in Europe and as fampridine sustained or modified release elsewhere), taken at 10 mg daily, as a treatment to improve walking in patients with multiple sclerosis (MS), as demonstrated by an increase in walking speed1. Approval for the treatment of MS patients was based on two phase 3 clinical trials that showed an approximate average 25% increase in walking speed among the 35% to 43% of patients who responded to treatment with dalfampridine-ER2,3.

The putative mechanism of action of dalfampridine in patients with MS is relief of the conduction block in demyelinated axons4; dalfampridine increases the ratio between the action current generated by the impulse and minimum amount of action current needed to maintain conduction for axonal conduction across demyelinated internodes5,6. Dalfampridine can also increase calcium influx at presynaptic sites, potentially enhancing neurotransmission in intact axons7,8, although the clinical relevance of these effects on neurotransmission is not known.

The pharmacokinetics of dalfampridine-ER have been evaluated in several human studies9–13 and single-dose pharmacokinetics show dose–plasma exposure proportionality9. Relative to immediate release dalfampridine, dalfampridine-ER was characterized by a longer time to maximum plasma concentration (3.2 vs 1.2 hours) and a longer apparent half-life (6.4 vs 3.7 hours), although peak plasma concentration and extent of exposure were higher in human subjects with renal impairment compared with healthy controls12. A more comprehensive review of the pharmacokinetics of dalfampridine has recently been published14, as well as a population pharmacokinetic study15.

In addition to characterization of a drug’s pharmacokinetic properties, identification of metabolites and their potential contribution to clinical efficacy and safety is important for understanding the overall pharmacodynamic effects of therapy. However, the complete metabolism of dalfampridine has only been partially characterized. An excretion mass-balance study evaluating the disposition of ^14^C-dalfampridine in humans showed that there is rapid and complete excretion by the urinary route, primarily as unchanged compound, and also detected the presence of two metabolites11. These metabolites, 2-hydroxy-4-AP and 3-hydroxy-4-AP, which quantitatively comprised 3% and 6%, respectively, of total radioactivity recovered from urine, were identified based on retention times using high performance liquid chromatography (HPLC) in the absence of appropriate reference standards. The objective of these studies is to provide an initial characterization of the metabolic profile of dalfampridine in two animal species that were used to support nonclinical toxicology studies.

## Methods

These studies, which evaluated and characterized the metabolism of 4-AP in rats and dogs, were performed in accordance with the Guide for the Care and Use of Laboratory Animals.

### Metabolic profiling in rats

Twelve adult male Sprague–Dawley rats were administered a single oral dose of ^14^C-4-AP, specific activity 6.55 μCi/mg (Amersham International PLC, Little Chalfont, UK) at 4.6 mg/kg. Portal and systemic blood samples were simultaneously collected from the hepatic portal vein and left cardiac ventricle, respectively, under anesthesia at 0.25, 0.5, 1, and 2 hours after ^14^C-4-AP administration from three animals at each time point. Blood samples were centrifuged, and the plasma was maintained at −20°C until analysis.

In addition to profiling the metabolites in each sample individually, two pooled plasma samples of both portal and systemic plasma from each animal were prepared, one representing the 0.25 and 0.5 hour time points, and the other representing the 1 and 2 hour time points. Aliquots of the individual (0.5 mL) and pooled (∼3.0 mL) plasma samples were freeze dried (Edwards Modulyo 4 K Freeze-Dryer; −50°C, 0.04 torr), and sequentially extracted with three aliquots of methanol by centrifugation. Supernatants were removed, pooled within samples, and evaporated at 37°C under nitrogen. The dry extracts were resuspended in 40 μL methanol and were mixed, sonicated (15 minutes), and centrifuged prior to chromatographic analysis.

Recovery of radioactivity in the methanol extract of each plasma sample was calculated by subtracting the amount of radioactivity in the residue from the total amount in the sample prior to extraction. Each residue was resuspended in distilled water (1.5 mL) and divided into two equal aliquots for liquid scintillation analysis. The amount of radioactivity in each plasma sample prior to extraction was also determined by liquid scintillation.

Extracts from individual and pooled samples were applied to thin layer chromatography (TLC) plates and eluted with a solvent consisting of dichloromethane, methanol, and concentrated ammonia solution in a volume ratio of 74:25:1. Co-chromatography was performed using unlabeled reference standards of 4-AP and 3-hydroxy-4-AP at concentrations of 2 mg/mL dissolved in methanol, as well as a representative diluted urine sample from a dog dosed with ^14^C-4-AP from the second canine study described above.

A Wallac 1409 automatic liquid scintillation analyzer (Pharmacia, Wallac Oy, Turku, Finland) was used to measure radioactivity of single-sample aliquots (50 μL) and resuspended residues resulting from the plasma extractions each diluted in MI-31 scintillation fluid (7 mL; Packard Instruments Ltd, Pangbourne, UK); quench correction curves were prepared using the spectral quench parameter of the external standard. Because of the relatively small quantities of radioactivity (<5000 dpm/sample), counting was preset at 10 minutes/10^4^ counts. Radioactivity less than twice the background was considered below the limit of quantitation (BLQ), and the maximum statistical counting error at this limit of detection was approximately 10% of the net count rate. The proportions of the radioactive components in each plasma sample were determined using Fuji BAS2000 Bio-Imaging Analyzer software.

### Metabolic profiling in dogs

The metabolic profile of 4-AP was evaluated in beagle dogs in two studies. The first canine study, performed by Hazleton Wisconsin, Inc. (Madison, WI, USA), included three male and three female beagles receiving a single 1 mg/kg oral dose of ^14^C-4-AP (2.0 mCi) in sterile water via gastric intubation. Urine samples were collected on ice at 0 to 6, 6 to 12, 12 to 24, 24 to 48, and 48 to 72 hours after administration, and the samples were frozen (−20°C) until analysis. A control sample was obtained from one of the animals prior to dosing.

At the time of analysis, samples were filtered (0.2 μm Gelman Acrodisc LC13 PVDF filters) prior to direct application of 5 g samples of urine onto a Biorad AG50W-X8 cation exchange column (7 × 2.5 cm; 200–400 mesh; H^+^ form), which was eluted and fractionated with successive applications of H_2_O (500 mL), 1 M NH_2_PO_4_ (500 mL), 3 M NH_4_H_2_PO_4_ (550 mL), 1 M NaCl (550 mL), 3 M NaCl (200 mL), and 5 M NaCl (200 mL). Each solvent fraction was collected separately for subsequent HPLC analysis and radioanalysis by liquid scintillation counting (LSC).

Identification of fractionated components was performed by HPLC analysis of selected fractions using Hewlett–Packard or Perkin–Elmer HPLC systems with a diode array or UV detection system at 254 nm. All analyses were performed isocratically on a Baker aliphatic sulfonic acid column (4.6 × 250 mm, 5 μm) with a mobile phase consisting of 90% 0.1 M KH_2_PO_4_ buffer (pH 3) and 10% methanol. Radioactivity was detected and quantitated using a Packard Flo-One/Beta Radiomatic Detector A500 or A250 with a 0.5 mL flow cell, a YtSi solid cell, or by collecting 1 minute fractions. Scintillation cocktail was added to each fraction for LSC, and counts <31,000 dpm were BLQ.

In the second canine study, performed by Huntingdon Life Sciences (Cambridgeshire, UK), a single oral dose of ^14^C-4-AP (1 mg/kg, specific activity 868 μCi/mg; Amersham International, Amersham, UK) was administered by gastric intubation to one male (11.3 kg) and one female (13.1 kg) dog who were both aged 1 year (Interfauna Ltd, Wyton, UK). Blood and urine samples were collected during the first 48 hours for determination of radioactivity. Blood was collected from a jugular vein into heparinized tubes at 0.5, 1, 1.5, 2, 3, 4, 5, 6, 12, 24, and 48 hours postdose, and the plasma was obtained by immediate centrifugation. Urine was collected into containers cooled by dry ice for the intervals of 0 to 6, 6 to 12, 12 to 24, and 24 to 48 hours postdose. The volume of each urine sample was measured, and in order to ensure complete recovery, the urine collection containers were rinsed with distilled water with the washings added to the sample. All samples were stored at −20°C until time of analysis.

After centrifugation to remove particulate matter, 25 to 50 μL aliquots of urine were applied to commercially prepared 0.25 mm thick silica gel TLC plates with a 60 F254 coating (E. Merck AG, Darmstadt, Germany). The mobile phase consisted of methanol and ammonium hydroxide (95:5, v/v). Samples were chromatographically separated along with authentic reference standards of ^14^C-4-AP, and unlabeled 2-hydroxy-4-AP and 3-hydroxy-4-AP. Non-radiolabeled standards were visualized under UV light. Radiolabeled components were visualized by placing the TLC plates in contact with a BAS2000 phosphoimaging plate (Fuji Photo Film Co Ltd, Japan), and the exposed plates were read with a Fujix BAS2000 Bio-Imaging Analyzer (Fuji Photo Film Co Ltd, Japan).

The profile of radioactivity in each eluted sample was obtained using a Berthold TLC Linear Analyzer (Model LB2842 and LB2832; Laboratorium Prof. Dr. Berthold, Wildbad, Germany). The positions of regions of interest corresponding to radioactive peaks were entered into the linear analyzer data system and the numerical values of the radioactivity were derived by the analyzer software. The proportion of each component was defined as the amount of radioactivity in the region of interest expressed as a percentage of the total radioactivity in the chromatogram. Authentic reference standards included ^14^C-4-AP, and unlabeled 2-hydroxy-4-AP, 3-hydroxy-4-AP, and 4-AP-N-oxide.

HPLC radiochromatographic profiles were also obtained for urine samples (50 to 200 μL, centrifuged to remove particulates) using isocratic conditions at room temperature using a μBondpak C_18_ column (Millipore 300 × 3.9 mm, 10 μm; Waters Ltd, Hertfordshire, UK) preceded by a μBondpak C_14_ guard-pak pre-column (Waters Ltd, Hertfordshire, UK) and a U6K Millipore injector (Waters Ltd, Hertfordshire, UK). Radiolabel and UV detection (263 nm) was performed using β-RAM instrumentation (Raytest Instrument Ltd, Sheffield, UK) and a UV2000 (Thermo Separation Products, Hertfordshire UK), respectively. The mobile phase (pH 3.5) consisted of KH_2_PO_4_ (10 mM), H_3_PO_4_ (10 mM), heptanesulphonic acid (0.15 mM), tetrabutyl ammonium iodide (2 mM), and acetonitrile (3.5%). Regions of interest on each radiochromatogram were entered into a LabChrom data system, and numerical values were derived by the software. The proportion of each component was defined as the area of the region of interest expressed as a percentage of the total area of all regions of interest in the radiochromatogram.

Enzymatic hydrolysis of urine samples was performed by incubation of 200 μL aliquots with an equal volume of β-glucuronidase type H-1 (EC 3.2.1.31; 1000 U/mL), which contains both glucuronidase and sulfatase activity, for approximately 17 hours at 37°C. Similarly, acid and alkaline hydrolysis were performed by adjusting 1 mL aliquots to pH 2 with HCl and pH 10 with NaOH, and incubating at 37°C for 5 hours. Additional aliquots (200 μL) of the 0 to 6 hour samples from the male dog were incubated with 20 μL of sulfatase (EC 3.1.6.1; 14.5 U/mL) for 17 hours and 65 hours at 37°C to further evaluate the samples for the presence of sulfate conjugate metabolites. All hydrolyzed samples were subsequently profiled by TLC.

Gas chromatography mass spectrometry (GC-MS) was used to identify the components further. Samples were prepared by scraping the relevant regions of the TLC plates followed by extraction with methanol and evaporation to dryness prior to trimethylsilyl derivatization. A VG 707E mass spectrometer (VG Analytical, Wythenshawe, UK) equipped with Hewlett Packard (Bracknell, UK) model 5790 gas chromatograph housing a DB5 fused silica capillary column (15 m × 0.25 mm, J&W Scientific, Folsom, CA, USA), and a VG 11-250 data system were used for the analyses. The split/splitless injector of the GC was held at 200°C and was operated in the splitless mode, the split valve being opened 30 seconds after injection of 1 to 2 μL sample volume. The mass spectrometer was operated in electron impact ionization mode with an electron energy of 70 eV and a trap current of 100 μA; ion source temperature was 200°C. After a 1 minute solvent delay, a mass spectrum was acquired every second during the run using a scan range of m/z 50-50, a scan rate of 0.8 seconds/decade, and an interscan time of 0.2 seconds. Electrospray ionization mass spectrometry (ESI) was performed using a model 5555-22 syringe pump (Harvard Apparatus, South Natick, MA, USA) connected to a model TWQ7000 mass spectrometry/mass spectrometry (MS/MS) instrument (Finnigan MAT, San Jose, CA, USA) via the ESI interface. A sample of approximately 10 μg in 250 μL methanol:water (1:1) was infused at a rate of 10 μL/minute. Nitrogen was used as a sheath gas at a head pressure of 70 psi and as an auxiliary gas at a flow rate of 500 mL/minute. A negative ion ESI was recorded by averaging several Q1 scans (mass range = m/z 80-700, scan rate of 3 seconds/scan) during sample infusion.

## Results

### Metabolic profile of 4-AP in rats

**Figure 1.**
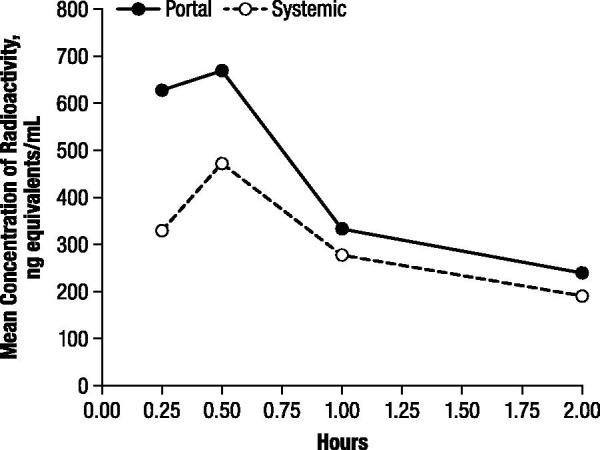
Mean concentration of radioactivity in portal and systemic rat plasma after oral administration of 4.6 mg/kg ^14^C-4-aminopyridine to male rats.

There was nearly complete recovery of total radioactivity in plasma extracts from portal and systemic blood. Although radioactivity was higher in portal plasma than in systemic plasma at all time points, maximal plasma concentrations were observed at 0.5 hours after administration, with means of 668.9 and 470.9 ng-equivalents/mL for portal and systemic plasma, respectively, decreasing to 239.8 and 189.8 ng-equivalents/mL, respectively, at 2 hours postdose (Figure 1).

Five radioactive components, M1–M5, were detected in both portal and systemic plasma. Components M1 and M2, from the pooled 0.25 and 0.5 hour samples and the pooled two 1 and 2 hour samples, coseparated with the reference standards of 3-hydroxy-4-AP and 4-AP, respectively; the typical retention fraction (*R_f_*) values for authentic reference standards were 0.10 for 3-hydroxy-4-AP and 0.35 for 4-AP, and the *R_f_* values for M1 and M2 were 0.09 and 0.28, respectively. Additionally, components M1, M2, and M3 coseparated with the same components isolated from the urine of a dog dosed with ^14^C-4-AP and identified as 3-hydroxy-4-AP, 4-AP, and 3-hydroxy-4-AP sulfate, respectively.

**Figure 2.**
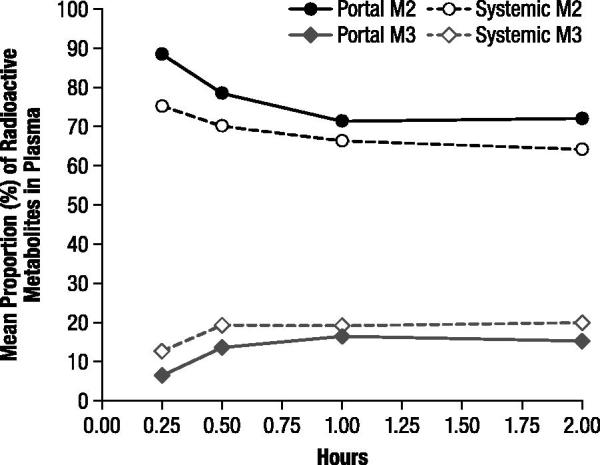
Mean proportions of radioactive components M2 and M3 in portal and systemic plasma, determined by thin layer chromatography and expressed as percentage of extract radioactivity, after oral administration of 4.6 mg/kg ^14^C-4-aminopyridine to male rats. M2 corresponded to unchanged 4-aminopyridine and M3 to 3-hydroxy-4-aminopyridine sulfate.

Most of the radioactivity detected in both portal and systemic plasma at each time point was associated with M2 and corresponded with unchanged 4-AP. The mean proportions of 4-AP ranged from 71.2% to 88.3% in portal plasma and from 64.2% to 75.1% in systemic plasma with the proportions generally decreasing over the 2 hour evaluation period (Figure 2). The maximal mean concentrations were 553.9 ng-equivalents/mL in portal plasma and 33.14 ng-equivalents/mL in systemic plasma, and the estimated area under the curve (*AUC*) of unchanged 4-AP for the 2 hour period was 597.7 and 383.9 ng-equivalents·h/mL, respectively. The 3-hydroxy-4-AP sulfate (M3) accounted for a notable proportion of radioactivity, and the proportions of this metabolite, which were consistently higher in the systemic samples, increased in both portal and systemic plasma samples from 0.25 to 0.5 hours and then remained relatively stable (Figure 2); the maximal mean concentrations of M3 were 89.5 and 89.8 ng-equivalents/mL in the portal and systemic plasma, respectively.

The proportions of radioactivity that corresponded with 3-hydroxy-4-AP (M1) and an unidentified component M4 were consistently low (≤3.2%), and were generally slightly higher in systemic plasma relative to portal plasma. Although a fifth component (M5) was detected in most samples, M5 was present at levels that were BLQ and thus could be neither identified nor quantified accurately.

### Metabolic profile of 4-AP in dogs

In the first dog study, there was 75% and 92% recovery of radioactivity in the urine by 12 hours in the male and female dogs, respectively. During the 12 to 24 hour period, an additional 6% was recovered in the males. However, because the lower limit of quantitation for LSC was 31,000 dpm, female urine samples collected 12 to 24 hours postdose and male urine samples collected after 24 hours postdose were not analyzed since they were BLQ.

**Figure 3.**
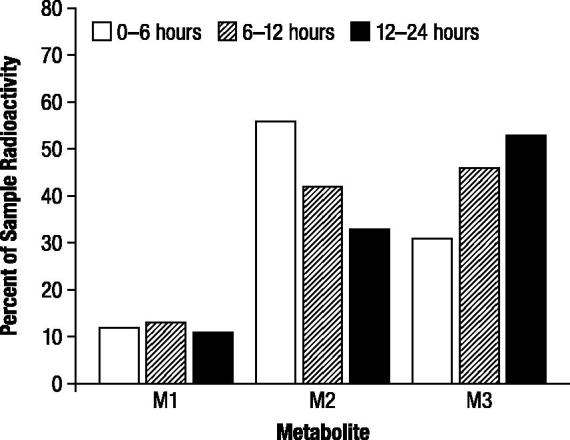
Mean proportions of radioactive components in urine determined by high performance liquid chromatography, expressed as percentage of sample radioactivity, after oral administration of 1 mg/kg ^14^C-4-aminopyridine to male (*n* = 3) and female (*n* = 3) beagle dogs. M1, M2, and M3 were identified as 3-hydroxy-4-aminopyridine, unchanged 4-aminopyridine, and 3-hydroxy-4-aminopyridine sulfate, respectively.

The proportions of radioactive components recovered in urine for each evaluable time period, expressed as the percentage of administered dose (Figure 3), indicated the presence of three major radioactive components (M1, M2, and M3). The relative proportions of M1 remained nearly constant throughout the collection periods, while M2 decreased as the relative proportion of M3 increased (Figure 3). No gender differences were observed in the study.

HPLC profiling demonstrated that the retention times of M1 and M2 were 10.1 and 12.7 minutes, respectively, corresponding to retention times for authentic standards of 10.0 minutes for 3-hydoxy-4-AP (M1) and 12.7 minutes for 4-AP (M2). The retention time for M3, 3.4 minutes, did not correspond to any of the standards used, and ESI was not able to provide positive identification of this component.

**Figure 4.**
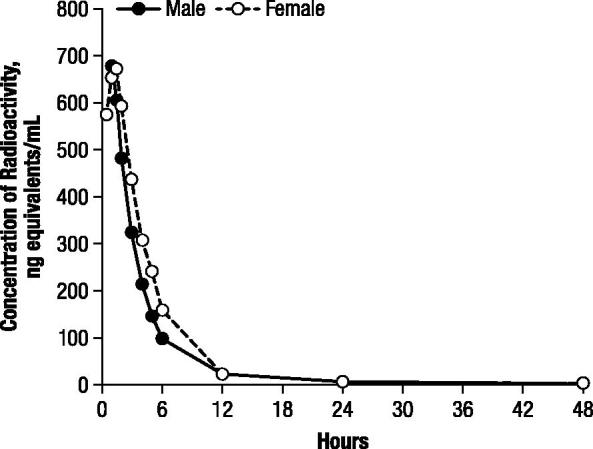
Concentrations of radioactivity in canine plasma after oral administration of 1 mg/kg ^14^C-4-aminopyridine to a male and female beagle dog.

In the second dog study, maximal plasma concentrations of radioactivity after oral administration of 1 mg/kg ^14^C-4-AP were observed at 1 hour in the male dog (676.3 ng-equivalents/mL) and 1.5 hours in the female dog (670.8 ng-equivalents/mL; Figure 4). Subsequent to achieving maximal concentrations, the plasma levels of radioactivity declined in a polyexponential manner (Figure 4).

Recovery of radioactivity in the urine was 77.6% and 93.2% in the male and female dogs, respectively; the low recovery in the male was a result of low urinary volume during the 6 to 12 hour time period. In both dogs, most of the radioactivity was excreted within the first 24 hours, with only 2.2% excreted between 24 and 48 hours.

**Figure 5.**
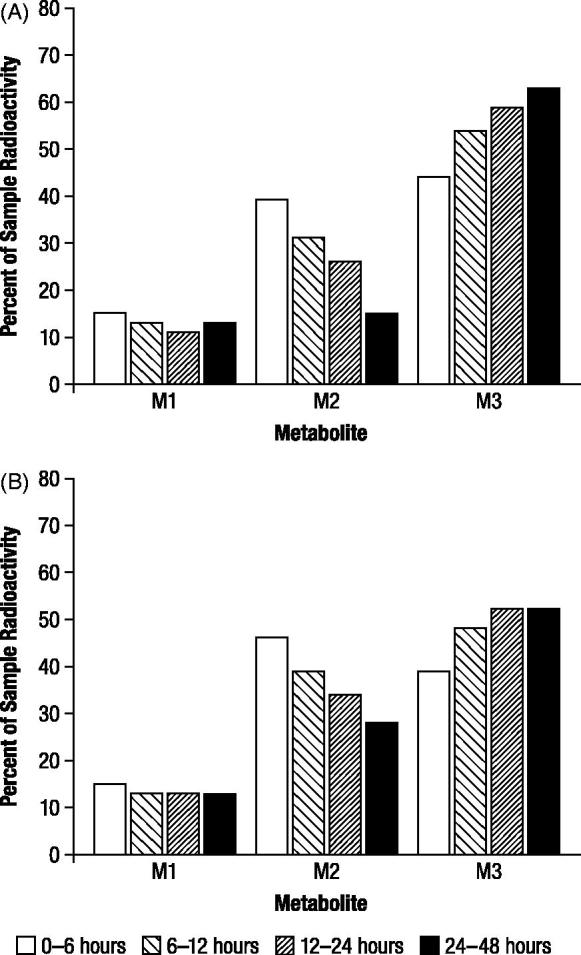
Mean proportions of radioactive components in urine determined by thin layer chromatography (A) and high performance liquid chromatography (B) expressed as percentage of sample radioactivity, after oral administration of 1 mg/kg ^14^C-4-aminopyridine to a male and female beagle dog. M1, M2, and M3 were identified as 3-hydroxy-4-aminopyridine, unchanged 4-aminopyridine, and 3-hydroxy-4-aminopyridine sulfate, respectively.

The proportions of radioactive components recovered in urine, expressed as the percentage of administered dose, were generally in good agreement between TLC and HPLC (Figure 5), and indicated the presence of three major radioactive components (M1, M2, and M3). M2 and M3 accounted for substantial proportions of urinary radioactivity during the first assessment interval (0 to 6 hours; Figure 5). However, while the proportion of M2 subsequently decreased in each successive assessment interval, the proportion of M3 increased; the proportions of M1 showed little change over the collection period. M3 was the major component excreted over the 48 hour assessment period (43%), and the proportion was approximately 1.5 times higher than M2 (29%) and 4 times higher than M1 (11%), as indicated by HPLC.

Use of hydrolysis to further characterize the metabolites and their relationship in the urine showed that the proportions of M1–M3 did not substantially change after incubation with β-glucuronidase relative to untreated urine (data not shown). Similarly, acid and alkaline hydrolysis had little effect on the proportions of the components (data not shown). However, incubation with sulfatase resulted in an increase in the proportion of component M1 (Table 1), from 15% to 32% after 17 hours of incubation, with a concomitant decrease in M3 from 36% to 23%; and this effect was more pronounced with a longer incubation time (Table 1). In contrast, the proportions of components in the untreated sample were similar after 17 and 65 hours of incubation (Table 1).

**Table 1.** Mean proportions of radioactive components in urine after oral administration of 1 mg/kg ^14^C-4-aminopyridine to a male and a female beagle dog determined by thin layer chromatography before and after sulfatase hydrolysis, expressed as percentage of sample radioactivity. Results are for samples collected during the 0–6 hours after administration.

Component	Proportion of sample radioactivity, %			
	17 hour incubation		65 hour incubation	
	Untreated	Sulfatase- treated	Untreated	Sulfatase- treated
Origin	2	3	2	3
M1	15	32	13	44
M2	46	41	42	45
M3	36	23	42	5

On TLC, components M1, M2, and M3 eluted with respective *R_f_* values of 0.41, 0.57, and 0.63 to 0.81. Co-chromatography identified M1 as 3-hydroxy-4-AP and M2 as unchanged 4-AP based on *R_f_* values of 0.46 and 0.59 for authentic reference standards, respectively. Similarly, HPLC retention times of 9 to 10 minutes for M1 and 7 to 8 minutes for M2 corresponded to authentic standard retention times of 9.5 and 7 minutes for 3-hydroxy-4-AP and M2 as unchanged 4-AP, respectively.

**Figure 6.**
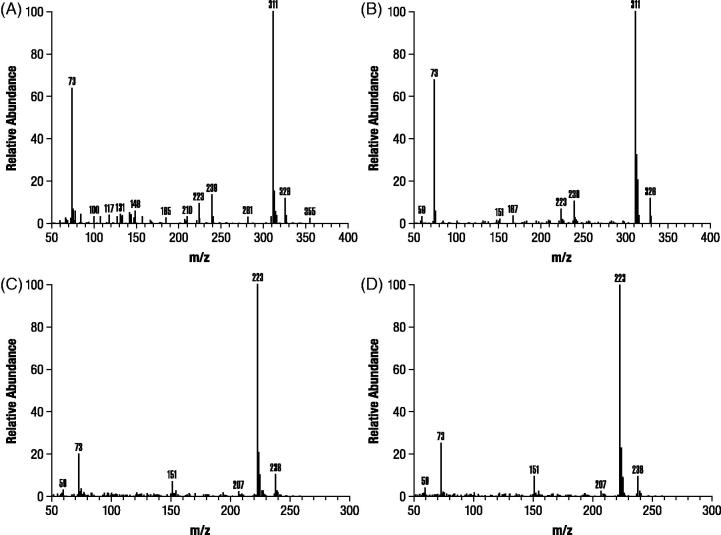
Mass spectra identification of components M1 and M2 isolated from dog urine in the second canine study. Identification of M1 (A) was based on authentic 3-hydroxy-4-aminopyridine (B) and M2 (C) was based on authentic 4-aminopyridine (D). Samples were derivatized prior to gas chromatography mass spectroscopy as stated in Methods.

**Figure 7.**
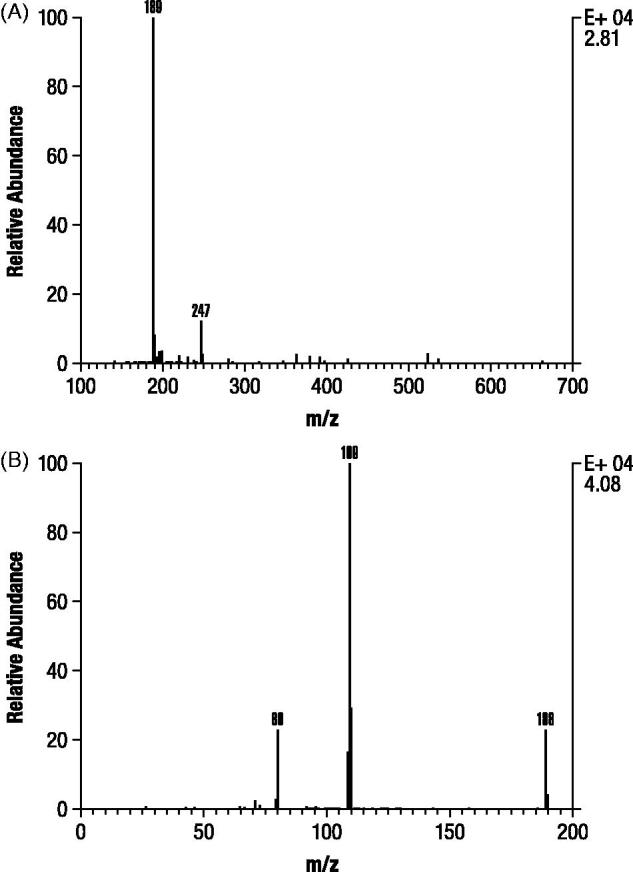
Mass spectra evaluation of M3 isolated from dog urine in the second canine study. Precursor electrospray ionization spectrum (A). Product ion scan of m/z 189 ion of component M3 (B).

Analysis of M1 and M2 by GC-MS after derivatization resulted in spectra that were identical with trimethylsilylated derivatives of authentic 3-hydroxy-4-AP and M2 as unchanged 4-AP, respectively (Figure 6). In contrast, M3 formed a dimethylsilyl derivative that only proceeded to a trimethylsilyl derivative after prolonged incubation (>24 hours) at room temperature, consistent with the probability that M3 is a conjugate. A subsequent precursor ion scan after negative ESI ionization of M3 resulted in a signal indicating an intense precursor ion at m/z 189 (Figure 7A), suggesting that M3 was 80 Daltons heavier than 3-hydroxy-4-AP as might be expected for a sulfate conjugate. A product ion scan obtained by fragmenting the m/z 189 ion (Figure 7B) showed that the molecule appeared to cleave almost entirely into two parts consisting of deprotonated 3-hydroxy-4AP (m/z 109) and 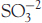 (m/z 80).

## Discussion

The studies reported here are consistent in showing that, in both dogs and rats, there is rapid absorption of 4-AP after oral administration and limited metabolism. In the rat study, unchanged 4-AP was the primary component recovered from portal and systemic plasma, and the data suggest that approximately 36% of the parent drug was removed by hepatic first-pass metabolism within 2 hours of administration. Approximately 22% of this amount was converted to metabolites, of which four were detected in both portal and systemic plasma in addition to unchanged 4-AP. Although the concentrations of two metabolites were too low for adequate quantitation and identification, the other two were identified as 3-hydroxy-4-AP and its sulfate conjugate. During the 2 hour sample-collection time period, only 3-hydroxy-4-AP sulfate, as measured in the systemic plasma, accounted for more than 10% of the total radioactivity (∼12% to 19.0%). The proportions of 3-hydroxy-4-AP sulfate in systemic plasma exceeded that in portal plasma at each time point, suggesting that both phase 1 (hydroxylation) and phase 2 metabolism (sulfate conjugation) occur in the liver.

The canine studies showed that after absorption, rapid and almost total excretion of the parent and metabolites occurs via renal excretion, with most of the radioactivity recovered by 24 hours. While a substantial proportion of 4-AP was excreted as unchanged parent compound (∼42%) during the first 6 hours, the presence of two metabolites was detected in dog urine, and these corresponded to the two metabolites observed at the highest concentrations in rat plasma.

Rapid urinary excretion and the presence of unchanged 4-AP in dog urine is consistent with a previously published canine study in which 60% of the administered dose was excreted in the urine as unchanged 4-AP by 10 hours postdose, with only 42% excreted by 6 hours16. Furthermore, these results are also in accord with human pharmacokinetic studies that reported rapid urinary excretion of 4-AP, albeit the proportion of unchanged compound was higher (approximately 90%) in the human studies11,17,18.

Both dog studies demonstrated the presence of 3-hydroxy-4-AP (M1), which accounted for approximately 13% of excreted radioactivity, and a second compound, M3, that was the predominant metabolite. This compound did not coelute by HPLC with any of the standards tested in the first study (3-hydroxy-4-AP, 4-AP, 4-AP-N-oxide, 1-methyl-4-piperidine, and 4-amino-2-pyridone), and thus was only tentatively determined to be a metabolite of 4-AP containing one additional oxygen atom and representing 26% to 37% of the total administered dose. However, in the second dog study, the additional GC-MS analyses performed to identify M3 strongly suggested that this metabolite is the sulfate conjugate of 3-hydroxy-4-AP (M1). The putative identification of M3 as 3-hydroxy-4-AP-sulfate was further supported by an observed decrease in M3 and concomitant increase in M1 after sulfatase hydrolysis, although conversion to the desulfated product was slow, requiring 65 hours incubation for almost complete conversion. Additionally, there was no evidence of hydrolysis after treatment with β-glucuronidase type H1, which contains sulfatase activity, or after acid and alkaline hydrolysis.

The identification of 3-hydroxy-4-AP as a metabolite of 4-AP in these rat and dog studies is consistent with its excretion as a presumptive human metabolite in a previously published excretion balance study11. The presence of 3-hydroxy-4-AP sulfate in rat plasma and dog urine contrasts with the above human study where a second metabolite, possibly 2-hydroxy-4-aminopyridine, was found in urine. This discrepancy may be accounted for by the fact that the human metabolites were identified based only upon approximate retention times using HPLC, and that a reference standard for 3-hydroxy-4-AP sulfate was not available.

## Conclusions

Following oral dosing, 4-AP was rapidly absorbed in both rats and dogs, and in dogs excretion was also rapid with almost complete urinary recovery. In both rats and dogs, the primary metabolites of 4-AP were identified as 3-hydroxy-4-AP and 3-hydroxy-4-AP sulfate. Based on identification of these metabolites, systemic clearance of 4-AP not accounted for by the renal excretion of the parent drug can be hypothesized to occur by liver metabolism in a two-step process that includes hydroxylation of 4-AP to 3-hydroxy-4-AP followed by sulfate conjugation to 3-hydroxy-4-AP sulfate. Further identification of 4--AP metabolites in humans and characterization of the pathways responsible for the limited amount of 4-AP metabolism will enable evaluation of the potential contribution of these metabolites to the overall pharmacodynamic effects of 4-AP treatment.

## Transparency

### Declaration of funding

This study was funded by Acorda Therapeutics Inc., Ardsley, NY, USA.

### Declaration of financial/other relationships

A.C., A.B., and T.J.P. are employees and stockholders of Acorda Therapeutics Inc., Ardsley, NY, USA.

## Acknowledgments

The authors wish to thank E.J. Bienen, PhD, of The Curry Rockefeller Group, LLC, Tarrytown, NY, USA for medical editorial assistance with this report. Editorial support was funded by Acorda Therapeutics Inc.

The dog studies were conducted at Huntingdon Life Sciences Ltd (Cambridgeshire, England) and Hazelton Wisconsin Inc. (Madison, WI, USA). The rat study was conducted at Huntingdon Life Sciences Ltd.
